# Paternal programming of breast cancer risk in daughters in a rat model: opposing effects of animal- and plant-based high-fat diets

**DOI:** 10.1186/s13058-016-0729-x

**Published:** 2016-07-26

**Authors:** Camile Castilho Fontelles, Luiza Nicolosi Guido, Mariana Papaléo Rosim, Fábia de Oliveira Andrade, Lu Jin, Jessica Inchauspe, Vanessa Cardoso Pires, Inar Alves de Castro, Leena Hilakivi-Clarke, Sonia de Assis, Thomas Prates Ong

**Affiliations:** 1 Department of Food and Experimental Nutrition, Faculty of Pharmaceutical Sciences, University of São Paulo, Avenida Professor Lineu Prestes 580, Bloco 14, São Paulo, SP 05508-000 Brazil; 2 Georgetown Lombardi Comprehensive Cancer Center, Washington, DC 20007 USA; 3 Food Research Center (FoRC), São Paulo, 05508-000 Brazil

**Keywords:** Paternal diet, Breast cancer, High-fat diet, Female offspring

## Abstract

**Background:**

Although males contribute half of the embryo’s genome, only recently has interest begun to be directed toward the potential impact of paternal experiences on the health of offspring. While there is evidence that paternal malnutrition may increase offspring susceptibility to metabolic diseases, the influence of paternal factors on a daughter’s breast cancer risk has been examined in few studies.

**Methods:**

Male Sprague-Dawley rats were fed, before and during puberty, either a lard-based (high in saturated fats) or a corn oil-based (high in n-6 polyunsaturated fats) high-fat diet (60 % of fat-derived energy). Control animals were fed an AIN-93G control diet (16 % of fat-derived energy). Their 50-day-old female offspring fed only a commercial diet were subjected to the classical model of mammary carcinogenesis based on 7,12-dimethylbenz[a]anthracene initiation, and mammary tumor development was evaluated. Sperm cells and mammary gland tissue were subjected to cellular and molecular analysis.

**Results:**

Compared with female offspring of control diet-fed male rats, offspring of lard-fed male rats did not differ in tumor latency, growth, or multiplicity. However, female offspring of lard-fed male rats had increased elongation of the mammary epithelial tree, number of terminal end buds, and tumor incidence compared with both female offspring of control diet-fed and corn oil-fed male rats. Compared with female offspring of control diet-fed male rats, female offspring of corn oil-fed male rats showed decreased tumor growth but no difference regarding tumor incidence, latency, or multiplicity. Additionally, female offspring of corn oil-fed male rats had longer tumor latency as well as decreased tumor growth and multiplicity compared with female offspring of lard-fed male rats. Paternal consumption of animal- or plant-based high-fat diets elicited opposing effects, with lard rich in saturated fatty acids increasing breast cancer risk in offspring and corn oil rich in n-6 polyunsaturated fatty acids decreasing it. These effects could be linked to alterations in microRNA expression in fathers’ sperm and their daughters’ mammary glands, and to modifications in breast cancer-related protein expression in this tissue.

**Conclusions:**

Our findings highlight the importance of paternal nutrition in affecting future generations’ risk of developing breast cancer.

**Electronic supplementary material:**

The online version of this article (doi:10.1186/s13058-016-0729-x) contains supplementary material, which is available to authorized users.

## Background

Breast cancer is a global public health problem, with nearly 1.7 million new cases diagnosed in 2012, representing 25 % of all cancers in women worldwide [1]. Its incidence is projected to rise significantly over the next 20 years despite current efforts to prevent the disease [2]. Although the precise reason for this growth is still not clear, it has been suggested that modern women’s lifestyles, including postponing first pregnancy and having fewer children, can explain the increase [3].

Nutritional habits, such as adoption of Western dietary patterns, are also linked to increased breast cancer risk [4]. These patterns are characterized by low consumption of fruits and vegetables, increased energy intake, and decreased energy expenditure, leading to obesity, as well as increased intake of saturated fatty acids (SFA), n-6 polyunsaturated fatty acids (PUFA), and *trans*-fatty acids and decreased intake of n-3 polyunsaturated fats [5, 6]. While the majority of epidemiological studies on nutrition and breast cancer risk have been focused on women’s diet in adulthood, accumulating epidemiological and experimental evidence highlights early life experiences, including nutrition, as relevant factors for later breast cancer risk determination [7]. The developmental origins of this cancer have been considered predominantly from a maternal perspective, with emphasis placed on the impact of high fat or energy intake during gestation and lactation on female offspring mammary gland development and later breast cancer risk [8, 9].

Although males contribute half of the embryo’s genome, only recently has interest begun to be directed toward the potential impact of paternal experiences on the health of offspring [10]. While experimental studies have shown that paternal malnutrition may increase the susceptibility of offspring to metabolic dysregulation, obesity, and cardiovascular diseases [11, 12], the influence of paternal factors on daughter’s breast cancer risk has been examined in few studies. Among them, epidemiological studies show an association between higher paternal education level, older age, and smoking with increased rate of breast cancer in the daughters [13, 14].

Unlike the female production of germ cells that takes place predominantly in early life [15], male production of germ cells starts in utero, with mature sperm cells being produced throughout the entire reproductive life of the male [16]. Because spermatogenesis can be dramatically influenced by environmental factors, including malnutrition, obesity, and an exposure to toxic compounds, the father’s health during preconception is now acknowledged as a critical factor in the context of the developmental origins of health and disease [17]. In addition to embryogenesis, gametogenesis comprises intense epigenetic (DNA methylation, histone modification, and microRNA [miRNA or miR] expression) remodeling [18, 19]. Thus, epigenetically inherited increased disease risk could be transmitted through the female as well as the male germline [20]. Specific windows within which male gametes would be especially prone to environmentally elicited epigenetic deregulation include prepuberty and the reproductive phase [21].

Given the marked increase in dietary fat intake over the past three decades [22], as well as the notion that different kinds of dietary fats can lead to different health outcomes [23], we designed this study to investigate whether, in rats, consumption of high levels of animal- or vegetable-based fats by fathers would affect their daughters’ risk of breast cancer. We also investigated the underlying cellular and molecular mechanisms. We fed male Sprague-Dawley rats, before and during puberty, either a lard-based (high in SFA) or a corn oil-based (high in n-6 PUFA) high-fat diet (60 % of fat-derived energy). Control animals were fed a control AIN-93G diet containing soybean oil as a fat source (16 % of fat-derived energy). Male rats were mated with female rats that were consuming a commercial diet. We show that paternal consumption of these high-fat diets elicited opposing effects, with animal fat increasing and vegetable oil decreasing breast cancer risk in the offspring. These effects could be linked to alterations in miRNA expression in fathers’ sperm and their daughters’ mammary glands, as well as to modifications in breast cancer-related protein expression in this tissue. These novel data show that paternal high-fat diets influence their female offspring’s susceptibility to mammary cancer, with consumption of lard increasing and corn oil reducing daughters’ mammary cancer risk. Thus, paternal diet before conception sets a stage for a daughter’s risk of developing breast cancer.

## Methods

### Experimental design

This study was approved by the ethics committee on animal experiments of the Faculty of Pharmaceutical Sciences, University of São Paulo (protocol number CEUA/FCF/381). Twenty-one-day-old male rats were divided into three groups (*n* = 20 rats per group): control rats (those fed the control AIN-93G diet, with 16 % of total calories provided by lipids), lard-fed males (exposed to a high-SFA diet, with 60 % of total calories provided mainly from lard), and corn oil-fed males (exposed to n-6 PUFA diet, with 60 % of total calories provided mainly from corn oil). At 12 weeks of age, all male rats were switched to a chow diet and mated by housing one male with one female per cage. Pregnant female rats and their offspring consumed only commercial laboratory chow (Nuvital Nutrientes, Colombo, Brazil). Body weight and food intake were recorded two or three times per week.

### Determination of the diets’ lipid profiles

The lipid profiles of the control, lard, and corn oil diets were determined according to the methods published by the Association of Official Analytical Chemists [24]. Fatty acids were esterified to fatty-acid methyl esters according to the method reported by Hartman and Lago [25], and their composition was analyzed with a gas chromatograph (GC 17A/Class GC 10; Shimadzu, Kyoto, Japan) equipped with a flame ionization detector and a SUPELCOWAX® 10 fused silica capillary column (30 mm × 0.25 mm inner diameter; Sigma-Aldrich, St. Louis, MO, USA). The temperature was set at 170 °C, raised to 225 °C at a rate of 1 °C/minute, and held for 25 minutes. The temperatures of the vaporizer and detector were 250 °C and 270 °C, respectively. Helium was used as the carrier gas (1 ml/minute). Identification of the fatty acids was performed by comparison of the retention times with the standard mixture of fatty-acid methyl esters. Each determination was performed in duplicate using two different samples for each diet.

### Insulin tolerance test

The tests were performed at 0800 h after the rats were fasted for 12 h, according to the method described by Takada et al. [26]. The insulin load (75 mU/100 g body weight) was injected as a bolus, and the blood glucose levels were determined at 0, 3, 6, 9, 12, and 30 minutes after injection in male rats and their 50-day-old female offspring. The AUC was calculated according to the trapezoid rule [27].

### Mature spermatozoa collection and purification

Control diet and lard- and corn oil-fed male rats were killed once females were pregnant, and the caudal epididymis was dissected for sperm collection. The cauda and vas deferens from male rats were collected, punctured, and transferred to tissue culture dishes containing M2 medium (M2 medium with HEPES, without penicillin and streptomycin, sterile-filtered, suitable for rat embryo; Sigma-Aldrich), where it was incubated for 1 h at 37 °C. Spermatozoa samples were washed with PBS and then incubated with somatic cell lysis buffer (SCLB; 0.1 % SDS, 0.5 % Triton X-100 in diethylpyrocarbonate water) for 1 h, according to a protocol described by Goodrich et al. [28]. SCLB was rinsed off with two washes of PBS, and the purified spermatozoa sample (at least 95 % purity as assessed by microscopy) was pelleted and used for miRNA extraction.

### Determination of daily sperm production

The right testis was maintained at −20 °C until processing to determine the daily sperm production. The technique proposed by Robb et al. [29] is based on the resistance of elongated spermatids present in phases 17–19 of spermatogenesis to intense mechanical stress due to the high compaction of chromatin.

### Sperm morphological analyses

According to the method of Seed et al. [30], the epididymis was previously frozen at −20 °C, underwent incision, and was subsequently immersed in PBS to promote the dissemination of gametes to the aqueous medium. Then the obtained solution was placed on slides for examination by light microscopy. Two hundred sperm per animal were analyzed microscopically at × 400 magnification.

### Mammary gland harvesting

Abdominal mammary glands of female offspring of control diet- and lard- and corn oil-fed male rats (*n* = 6 per group) were collected on postnatal day 50 and used for preparing mammary whole mounts and miRNA and protein extraction.

### Analysis of mammary gland morphology and development

Whole-mount preparations of the fourth abdominal mammary gland from 50-day-old female offspring (*n* = 5/group) were obtained, and the epithelial elongation and number of terminal end buds (TEBs) were determined as described by de Assis et al. [31].

### Mammary tumor induction

Mammary tumors were induced in 50-day-old female rat offspring (*n* = 24 rats/group) by administration of 7,12-dimethylbenz[a]anthracene (DMBA, 50 mg/kg body weight; Sigma-Aldrich). The carcinogen was dissolved in corn oil and administered by oral gavage. Animals were examined for mammary tumors by palpation twice per week. Latency of tumor appearance, the number of animals with tumors, and the number of tumors per animal (multiplicity) were evaluated. The tumor volume was calculated with tumor measures of length (*a*), height (*b*), and width (*c*) taken with a caliper rule once per week since tumor appearance and throughout the experiment. The formula (1/6 × 3.14) × (*a* × *b* × *c*) was used to calculate the tumor volume, as described by Spang-Thomsen et al. [32]. The tumor growth rate was calculated using the measured volumes of each tumor at a given week (*d*) and the subsequent week (*e*) of appearance using the formula [(*e* − *d*)/*d*] × 100. Those animals in which tumor burden approximated 10 % of total body weight were killed. All others animals were killed 19 weeks after carcinogen administration.

### Analysis of mammary gland and tumor cell proliferation and apoptosis in female offspring

Cell proliferation was evaluated in mammary glands (ducts and lobules) and tumors from 50-day-old female offspring (*n* = 4/group) by Ki-67 immunohistochemistry. After being harvested, mammary tissue was directly fixed in 10 % buffered formalin, embedded in paraffin, and sectioned. Sections were then deparaffinized in xylene and hydrated through graded ethanol. Antigen retrieval was performed with 10 mM citrate buffer, pH 6, for 20 minutes in a pressure cooker. Peroxidase blocking was performed with 10 % H_2_O_2_ for 10 minutes, and nonspecific binding was blocked for 1 h with 1 % skimmed milk in PBS. Sections were incubated overnight with anti-rat Ki-67 primary antibody (Abcam, Cambridge, UK) at a 1:50 dilution. After washes, sections were incubated with the LSAB 2 System-HRP kit (Dako, Carpinteria, CA, USA) according to the manufacturer’s instructions, stained with 3,3′-diaminobenzidine in chromogenic solution (Dako) for 10 minutes, washed, and counterstained for 1.5 minutes with hematoxylin. Cell proliferation was quantified by assessing the number of Ki-67-positive cells among 1000 cells. The slides were evaluated using ImageJ software (National Institutes of Health, Bethesda, MD, USA). Apoptosis analysis was conducted in mammary glands (ducts and lobules) and tumors from 50-day-old female offspring (*n* = 4/group), according to the method described by Elmore et al. [33], using ImageJ software. Results are presented as mean number of apoptotic cells per 1000 cells.

### microRNA expression profile analysis

Total RNA from paternal sperm and their female offspring’s total mammary gland was extracted using the miRNeasy Mini Kit (QIAGEN, Valencia, CA, USA) according to the manufacturer’s instructions. RNA samples were quantified and stored at −80 °C until use. miRNA arrays were performed at the Genomics and Epigenomics Shared Resources at Georgetown University using Applied Biosystems TaqMan Array Rodent MicroRNA arrays (Life Technologies, Carlsbad, CA, USA) to generate the miRNA expression profiles for each experimental group. The TaqMan® Array Rodent MicroRNA A + B Cards Set v3.0 is a two-card set containing a total of 384 TaqMan® MicroRNA assays per card (Life Technologies). The set enables accurate quantitation of 641 and 373 unique miRNAs for rat. There are three TaqMan® MicroRNA Assay endogenous controls for each species on each array to aid in data normalization [34]. The geNorm algorithm was applied to those endogenous controls to determine the optimal number of stable controls. The geometric mean of these selected controls was used for array normalization. To conduct further statistical analysis, the normalized value was log-transformed to meet the *t* test requirement. Statistical analysis was conducted using the limma package in R [35]. miRNAs that had a false discovery rate <0.1 were considered as significantly altered and selected for further analysis. Target prediction for miRNAs of interest was conducted using TargetScan (release 6.2). The predicted targeted messenger RNA (mRNA) list was then uploaded to Ingenuity Pathway Analysis (IPA; QIAGEN Silicon Valley, Redwood City, CA, USA) for gene set enrichment analysis. We selected the top canonical pathways for further analysis.

### Analysis of protein levels in mammary glands of female offspring

Protein levels of 50 ng/μl were assessed by Western blot analysis in total mammary glands obtained from 50-day-old female rats (*n* = 5 per group). Total protein was extracted from mammary tissues using radioimmunoprecipitation assay buffer with protease inhibitor (Roche, Basel, Switzerland), glycerophosphate (10 mM), sodium orthovanadate (1 mM), pyrophosphate (5 mM), and phenylmethylsulfonyl fluoride (1 mM). The samples were mixed with the buffer for 5 minutes, then incubated on ice for 30 minutes and centrifuged for 10 minutes at high speed. Protein in the supernatant was quantified using Pierce bicinchoninic acid protein assay reagent (Thermo Scientific, Rockford, IL, USA). Protein extracts were resolved on a 4–12 % gradient denaturing polyacrylamide gel (SDS-PAGE). Proteins were transferred using the Invitrogen iBlot® 7-Minute Dry Blotting System (Life Technologies) and blocked with 5 % nonfat dry milk for 1 h at room temperature. Membranes were incubated with the specific primary antibodies at 4 °C overnight. After several washes, the membranes were incubated with HRP-conjugated secondary antibody (1:5000; Santa Cruz Biotechnology, Dallas, TX, USA) at room temperature for 1 h. Membranes were developed using HyGLO chemiluminescent HRP antibody detection reagent (Denville Scientific Inc., Metuchen, NJ, USA), and exposed to Kodak autoradiography films (Carestream Health, Rochester, NY, USA). The optical density of the bands was quantified using Quantity One software (Bio-Rad Laboratories, Hercules, CA, USA). To control for equal protein loading, expression of the proteins of interest was normalized to the β-actin signal.

### Statistical analysis

Results are expressed as the mean ± SEM, and all analyses were conducted with the limma package in R. Multiple-group comparisons were performed using one-way analysis of variance (ANOVA) followed by a least significant difference (LSD) test, and two-group comparisons were performed using Student’s *t* test. Repeated-measures ANOVA was applied for caloric intake data evaluation, and Kaplan-Meier curves and log-rank tests were applied for determining differences in tumor incidence. For all data analyses, *p* ≤ 0.05 was applied as the threshold for statistical significance.

## Results

### Paternal dietary and health parameters

**Fig. 1:**
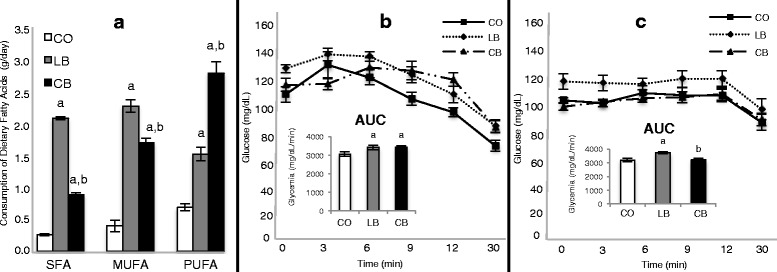
Paternal fatty-acid consumption and insulin tolerance test. **a** Male rats fed a control diet (CO) or a lard-based (LB) or corn oil-based (CB) high-fat diet consumption of dietary fats (saturated fatty acids [SFA], monounsaturated fatty acids [MUFA], and polyunsaturated fatty acids [PUFA]) (*n* = 20 per group). **b** Insulin tolerance test (ITT) of the CO-, LB-, and CB-fed male rats (*n* = 6 per group). *Inset*: ITT is shown as the AUC. **c** Fifty-day-old female offspring (*n* = 6 per group). *Inset*: ITT is shown as the AUC. Statistically significant difference (*p* ≤ 0.05) compared with ^a^CO and ^b^LB, according to analysis of variance followed by a least significant difference test. The data are expressed as mean ± SEM

Compared with control diet-fed male rats, the ones that were on the lard- or corn oil-based high-fat diets consumed more (*p* ≤ 0.05) SFA (predominantly palmitic [C16:0] and stearic [C18:0] acids), monounsaturated fatty acids (MUFA) (predominantly oleic acid [C18:1n9c]) and PUFA (predominantly linoleic acid [C18:2n6c]) (Fig. 1a). Corn oil-fed male rats consumed less (*p* ≤ 0.05) SFA (predominantly palmitic [C16:0] and stearic [C18:0] acids) and MUFA (predominantly oleic acid [C18:1n9c]) and more (*p* ≤ 0.05) PUFA (predominantly linoleic acid [C18:2n6c]) than the lard-fed male rats (Fig. 1a). Daily caloric intake was approximately 7 % higher (*p* ≤ 0.05) in both lard- and corn oil-fed male rats than in control diet-fed male rats (data not shown). Although lard- and corn oil-fed male rats consumed nearly the same amount of calories per day, lard-fed male rats gained more (*p* ≤ 0.05) weight than control diet- and corn oil-fed male rats (*p* ≤ 0.05) (Table 1). There was no difference (*p* > 0.05) between control diet- and corn oil-fed male rats regarding weight gain. Both lard- and corn oil-fed male rats had greater (*p* ≤ 0.05) abdominal, retroperitoneal, and epididymal fat pad weights than control diet-fed male rats (Table 1). Compared with lard-fed male rats, corn oil-fed male rats had lesser (*p* ≤ 0.05) epididymal fat pad weights, but there was no difference (*p* > 0.05) in abdominal or retroperitoneal fat pad weights (Table 1). Further, lard-fed male rats had lesser testicle (*p* ≤ 0.05), epididymis (*p* ≤ 0.05), and seminal vesicle (*p* ≤ 0.08) weights than the control diet- and corn oil-fed male rats (Table 1). There was no difference (*p* > 0.05) between control diet- and corn oil-fed male rats regarding these parameters (Table 1). Lard-fed male rats also had fewer (*p* ≤ 0.05) normal sperm cells and lower (*p* ≤ 0.05) daily sperm production than control diet- and corn oil-fed male rats (Table 1). There was no difference (*p* > 0.05) between control diet- and corn oil-fed male rats regarding these parameters (Table 1). Both lard- and corn oil-fed male rats had higher (*p* ≤ 0.05) fasting glucose levels than control diet-fed male rats (Table 1). There was no difference (*p* > 0.05) between lard- and corn oil-fed male rats regarding this parameter (Table 1). Further, in the insulin tolerance test, lard- and corn oil-fed male rats had higher (*p* ≤ 0.05) AUCs than control diet-fed male rats (Fig. 1b), indicating that they were insulin-intolerant. There was no difference (*p* > 0.05) between lard- and corn oil-fed male rats regarding this parameter (Fig. 1b).

**Table 1:** Health parameters of male rats and their 50-day-old female offspring in control diet, lard-based diet, and corn oil-based diet groups

	CO	LB	CB
Male rats			
Weight gain	363.1 ± 6.4 g	398.2 ± 12.0 g^a^	369.4 ± 7.8 g^b^
Fat weight			
Abdominal fat	1.4 ± 0.1 g/100 g body weight	2.7 ± 0.2 g/100 g body weight^a^	2.6 ± 0.2 g/100 g body weight^a^
Retroperitoneal fat	0.8 ± 0.1 g/100 g body weight	1.9 ± 0.1 g/100 g body weight^a^	1.7 ± 0.2 g/100 g body weight^a^
Epididymal fat	1.2 ± 0.1 g/100 g body weight	2.7 ± 0.1 g/100 g body weight^a^	2.0 ± 0.2 g/100 g body weight^a,b^
Reproductive organs			
Testicle	0.45 ± 0.01 g/100 g body weight	0.40 ± 0.01 g/100 g body weight^a^	0.45 ± 0.01 g/100 g body weight^b^
Epididymis	0.14 ± 0.00 g/100 g body weight	0.13 ± 0.00 g/100 g body weight^a^	0.14 ± 0.00 g/100 g body weight^b^
Seminal vesicle	0.33 ± 0.01 g/100 g body weight	0.29 ± 0.02 g/100 g body weight^c^	0.33 ± 0.02 g/100 g body weight^d^
Sperm morphology (% normal)	66.4 ± 2.8 %	50.1 ± 3.0 %^a^	70.9 ± 2.9 %^b^
Daily sperm production, *n*/testicle/day	31 ± 1.9 × 10^6^	23 ± 0.9^a^ × 10^6^	29 ± 0.8^b^ × 10^6^
Fasting glycemia	100.8 ± 3 mg/dl	124.6 ± 4 mg/dl^a^	116.1 ± 3 mg/dl^a^
50-day-old female offspring			
Birth weight	8.0 ± 1.5 g	8.8 ± 1.0 g^a^	9.1 ± 1.5 g^a^
Weight gain	138.9 ± 1.5 g	147.0 ± 1.2 g^a^	142.1 ± 1.1 g^b,c^
Retroperitoneal fat	0.9 ± 0.1 g/100 g body weight	1.1 ± 0.0 g/100 g body weight^a^	1.2 ± 0.1 g/100 g body weight^a^
Fasting glycemia	106.4 ± 2.0 mg/dl	112.5 ± 1.9 mg/dl^a^	106.3 ± 1.2 mg/dl^b^

*Abbreviations: CB* rats fed a corn oil-based high-fat diet and their offspring, *CO* rats fed a control diet and their offspring, *LB* rats fed a lard-based high-fat diet and their offspring

Statistically significant difference (*p* ≤ 0.05) compared with ^a^CO and ^b^LB, according to analysis of variance followed by least significant difference test. Marginal difference (*p* ≤ 0.08) compared with ^c^CO and ^d^LB, according to *t* test. The data are expressed as mean ± SEM (*n* = 20 per group)

### Female offspring health parameters

Female offspring of both lard- and corn oil-fed male rats had greater birth weight (*p* ≤ 0.05) and greater weight gain (*p* ≤ 0.05; *p* ≤ 0.08 for offspring of corn oil-fed male rats) than offspring of control diet-fed male rats (Table 1). There was no difference (*p* > 0.05) between female offspring of lard- and corn oil-fed male rats regarding birth weight (Table 1). Female offspring of corn oil-fed male rats had less (*p* ≤ 0.05) weight gain than offspring of lard-fed male rats (Table 1). Similarly to the male rats, female offspring of both the lard-fed and the corn oil-fed male rats had greater (*p* ≤ 0.05) retroperitoneal fat weights than offspring of control diet-fed male rats (Table 1). There was no difference (*p* > 0.05) between female offspring of lard- or corn oil-fed male rats regarding this parameter (Fig. 1c). Female offspring of lard-fed male rats had higher (*p* ≤ 0.05) fasting glucose levels (Table 1) and higher (*p* ≤ 0.05) AUCs than female offspring of control diet- or corn oil-fed male rats (Fig. 1c). There was no difference (*p* > 0.05) between female offspring of control diet- and corn oil-fed male rats regarding these parameters (Table 1 and Fig. 1c).

### Female offspring mammary gland morphology

**Fig. 2:**
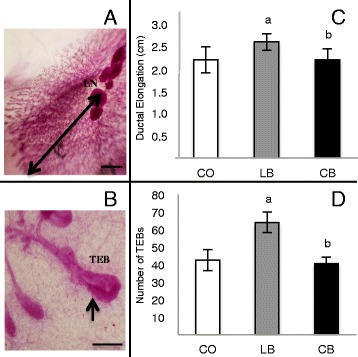
Mammary gland development of 50-day-old female offspring of control diet (CO)-, lard (LB)-, and corn oil (CB)-fed male rats. **a** Histological depiction of the fourth abdominal mammary gland showing ductal elongation, indicated by *arrow*. **b** Terminal end buds (TEBs), indicated by *arrows*. **c** Ductal elongation. **d** Number of TEBs. All values are expressed as the mean ± SEM. Statistically significant difference (*p* ≤ 0.05) compared with ^a^CO and ^b^LB, according to analysis of variance followed by a least significant difference test. The data are expressed as mean ± SEM (*n* = 5 per group)

Mammary gland morphology was assessed on the basis of mammary whole mounts obtained from 50-day-old female offspring. Both elongation of the mammary epithelial tree (Fig. 2c) and the number of TEBs (Fig. 2d) were higher (*p* ≤ 0.05) in female offspring of lard-fed male rats than in female offspring of control diet- and corn oil-fed male rats. There was no difference (*p* > 0.05) between female offspring of control diet- and corn oil-fed male rats regarding these parameters (Fig. 2c and d).

### Female offspring mammary gland tumors data

**Fig. 3:**
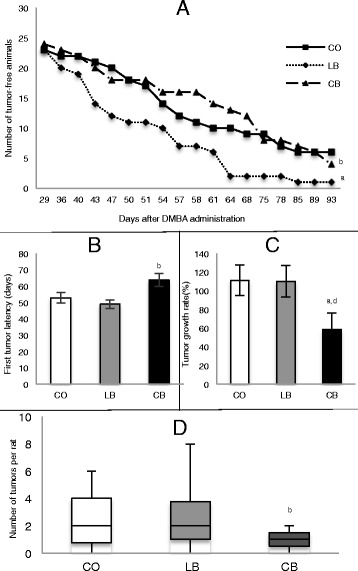
Mammary tumorigenesis in female offspring of control diet (CO)-, lard (LB)-, and corn oil (CB)-fed males. **a** Number of tumor-free rats. **b** Number of days before the appearance of the first tumor. **c** Tumor growth rate in the first week of appearance. **d** Total number of tumors per rat (multiplicity). Statistically significant difference (*p* ≤ 0.05) compared with ^a^CO and ^b^LB, according to analysis of variance followed by a least significant difference test. Marginal difference (*p* ≤ 0.08) compared with ^d^LB, according to *t* test. The data are expressed as mean ± SEM (*n* = 24 per group). *DMBA* 7,12-dimethylbenz[a]anthracene

Mammary tumors in the female offspring were induced by administering the carcinogen DMBA. Female offspring of lard-fed male rats had increased mammary tumor incidence (*p* ≤ 0.05) compared with offspring of both control diet- and corn oil-fed male rats (Fig. 3a). There was no statistical difference (*p* > 0.05) between female offspring of control diet- and corn oil-fed male rats regarding tumor incidence. Female offspring of corn oil-fed male rats exhibited longer (*p* ≤ 0.05) tumor latency and lower tumor multiplicity (*p* ≤ 0.05) than female offspring of lard-fed male rats (Fig. 3b and d). Compared with female offspring of control diet-fed male rats, female offspring of lard- and corn oil-fed male rats did not show differences (*p* > 0.05) regarding tumor latency and multiplicity. Further, female offspring of corn oil-fed male rats showed less (*p* ≤ 0.05) tumor growth in the first week of tumor appearance than offspring of both control diet- and lard-fed male rats (Fig. 3c). There was no statistical difference (*p* > 0.05) between offspring of control diet- and lard-fed male rats regarding tumor growth. Additionally, there was no statistical difference among groups in the tumor growth rate for the remaining experimental weeks.

### Female offspring mammary gland and tumor cell proliferation and apoptosis

**Fig. 4:**
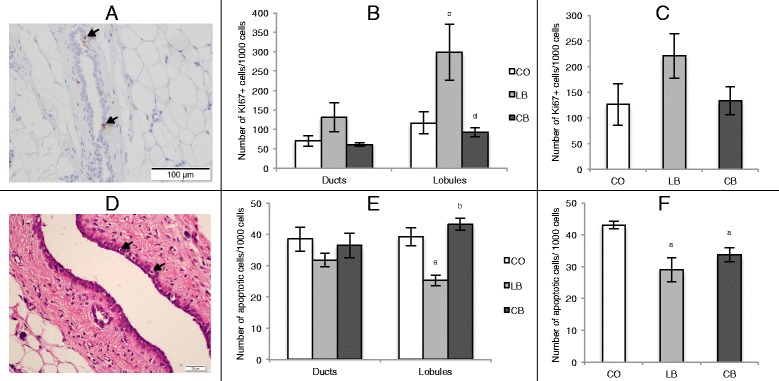
Quantification of cell proliferation and apoptosis in mammary gland and tumors. **a** Photomicrograph (×20 original magnification) of Ki-67 immunostaining (cells indicated by *arrows*). **b** Quantification of cell proliferation in the mammary gland ducts and lobules of 50-day-old female offspring of control diet (CO)-, lard (LB)-, and corn oil (CB)-fed male rats. **c** Quantification of cell proliferation in mammary tumors of female offspring from CO-, LB-, and CB-fed male rats. **d** Photomicrograph (×40 original magnification) showing apoptotic cells (indicated by *arrows*). **e** Quantification of apoptosis in the mammary gland ducts and lobules of 50-day-old female offspring of CO-, LB-, and CB-fed male rats. **f** Quantification of apoptosis in mammary tumors of female offspring of CO-, LB-, and CB-fed males. Statistically significant difference (*p* ≤ 0.05) compared with ^a^CO and ^b^LB, according to analysis of variance followed by a least significant difference test. Marginal difference (*p* ≤ 0.06) compared with ^c^CO and ^d^LB, according to *t* test. The data are expressed as mean ± SEM (*n* = 4 per group)

Female offspring of lard-fed male rats exhibited an increased (*p* ≤ 0.06) number of proliferative cells (Fig. 4b) and a decreased (*p* ≤ 0.05) number of apoptotic cells (Fig. 4e) in mammary gland lobules compared with female offspring of control diet- and corn oil-fed male rats. There was no difference (*p* > 0.05) between female offspring of control diet- and corn oil-fed male rats regarding these parameters (Fig. 4b and e). Further, there was no difference (*p* > 0.05) in cell proliferation and apoptosis in mammary gland ducts among female offspring of all groups. Female offspring of both lard- and corn oil-fed male rats exhibited a decreased (*p* ≤ 0.05) number of apoptotic cells (Fig. 4f) in mammary tumors compared with female offspring of control diet-fed male rats. There was no difference (*p* > 0.05) between female offspring of lard- and corn oil-fed male rats regarding this parameter (Fig. 4f). In addition, there was no difference (*p* > 0.05) among groups regarding cell proliferation in the mammary tumors (Fig. 4c).

### miRNA expression profile in fathers’ sperm cells and in their daughters’ mammary glands

**Fig. 5:**
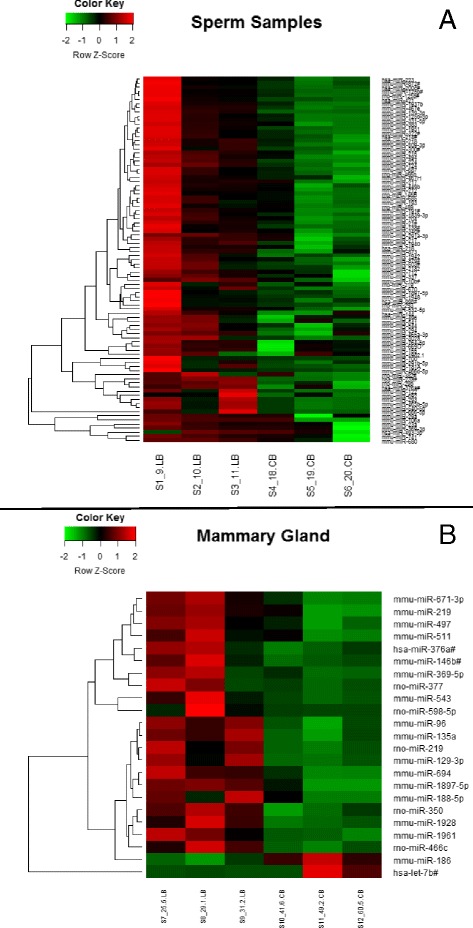
Heat map of microRNA (miRNA or miR) expression profiles. **a** Heat map of miRNA expression profile in sperm of the male rats from control diet-, lard-, and corn oil-fed male rats. **b** Heat map of miRNA expression profile in normal mammary tissue of 50-day-old female offspring (*n* = 3 per group)

To compare the outcomes of the distinct paternal high-fat diets on the basis of miRNA expression, Applied Biosystems TaqMan Rodent MicroRNA arrays were used to generate the miRNA profile for lard- and corn oil-fed fathers’ sperm cells, as well as for their respective daughters’ mammary glands. The microarray data are deposited in the Gene Expression Omnibus (GEO) public repository under accession number [GEO:GSE77012]. Corn oil-fed male rats had 89 downregulated (*p* ≤ 0.05) miRNAs in the sperm compared with lard-fed male rats (Fig. 5a). Furthermore, female offspring of corn oil-fed male rats had 21 downregulated (*p* ≤ 0.05) and 2 upregulated (*p* ≤ 0.05) miRNAs in their mammary glands compared with female offspring of lard-fed male rats (Fig. 5b). There were three miRNAs that were downregulated in both the sperm and the mammary glands of the corn oil-fed fathers and their daughters, respectively: miR-1897-5p, miR-219-1-3p, and miR-376a#. IPA (Additional file 1: Table S1) indicated that these miRNAs could regulate signaling pathways associated with key physiological processes such as growth hormone, phosphatase and tensin homolog (PTEN), and prolactin signaling, as well as disease processes such as Huntington’s disease, cardiac hypertrophy, type 2 diabetes mellitus, and breast cancer.

### Protein expression in female offspring mammary gland

**Fig. 6:**
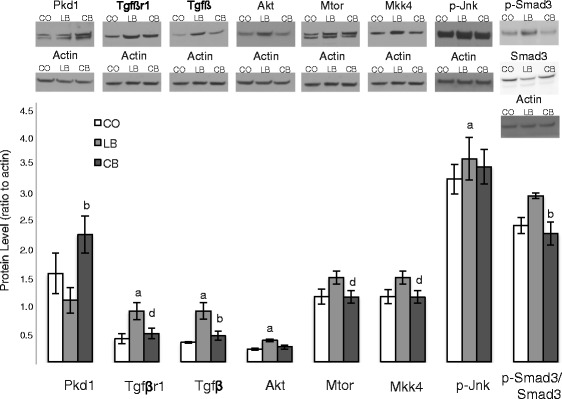
Protein alterations associated with microRNA (miRNA) expression. Western blot analysis of protein kinase D1 (Pkd1), transforming growth factor, beta receptor I (Tgfβr1), transforming growth factor beta (Tgfβ), v-akt murine thymoma viral oncogene (Akt), mechanistic target of rapamycin (Mtor), mitogen-activated protein kinase kinase 4 (Mkk4), phosphorylated mitogen-activated protein kinase 8 (p-Jnk), and phosphorylated Smad family member 3/Smad family member 3 (p-Smad3/Smad3) ratio protein expression in mammary glands of 50-day-old female offspring of control diet (CO)-, lard (LB)-, and corn oil (CB)-fed male rats. Statistically significant difference (*p* ≤ 0.05) compared with ^a^CO and ^b^LB, according to analysis of variance followed by a least significant difference test. Marginal difference (*p* ≤ 0.06) compared with ^d^LB, according to *t* test. The data are expressed as mean ± SEM (*n* = 5 per group)

Since miR-1897-5p, miR-219-1-3p, and miR-376a# can directly or indirectly modulate several targets (shown in Additional file 1: Table S1), we decided to perform Western blot analysis of the following proteins linked to breast cancer: CCAAT/enhancer-binding protein beta (Cebpβ), caspase 3 (Casp3), insulin-like growth factor 1 receptor (Igf1r), protein kinase D1 (Pkd1), and transforming growth factor, beta receptor I (Tgfβr1). On the one hand, there was no difference (*p* > 0.05) among female offspring of the control diet-, lard-, and corn oil-fed male rats regarding Cebpβ, Casp3, and Igf1r levels (data not shown). On the other hand, female offspring of the corn oil-fed male rats had higher (*p* ≤ 0.05) Pkd1 levels in the mammary glands than female offspring of lard-fed, but not control diet-fed, male rats (Fig. 6). There was no difference (*p* > 0.05) between female offspring of control diet- and lard-fed male rats regarding this protein (Fig. 6). In addition, Tgfβr1 levels were significantly increased in the offspring of lard-fed male rats (Fig. 6) compared with offspring of both control diet-fed (*p* ≤ 0.05) and corn oil-fed (*p* ≤ 0.06) male rats. There was no difference (*p* > 0.05) between female offspring of corn oil-fed and control diet-fed male rats regarding this protein (Fig. 6). Interestingly, both proteins are involved in regulating epithelial-to-mesenchymal transition (EMT): Pkd1 inhibits this process [36], and Tgfβr1 promotes it [37].

We further explored if Tgfβ and key regulators of its activity were altered by measuring protein levels of v-akt murine thymoma viral oncogene (Akt), cofilin (Cfl), v-raf leukemia viral oncogene (c-Raf), extracellular signal-regulated kinase 1/2 (Erk1/2), phosphorylated mitogen-activated protein kinase 8 (p-Jnk), mitogen-activated protein kinase 4 (Mkk4), mechanistic target of rapamycin (Mtor), mitogen-activated protein kinase 14 (p38), phosphorylated Smad family member 3/Smad family member 3 (p-Smad3/Smad3) ratio, and Harvey rat sarcoma virus oncogene (Ras). Tgfβ protein expression was higher (*p* ≤ 0.05) in the mammary glands of the female offspring of lard-fed male rats than in the offspring of control diet- and corn oil-fed male rats. There was no difference (*p* > 0.05) between female offspring of corn oil- and control diet-fed male rats regarding this protein (Fig. 6). Further, the levels of Akt and p-Jnk were higher (*p* ≤ 0.05) in the female offspring of lard-fed male rats than in female offspring of control diet-fed male rats (Fig. 6). There was no difference (*p* > 0.05) in female offspring of corn oil-fed male rats and the offspring of control diet- and lard-fed male rats regarding these proteins (Fig. 6). Female offspring of corn oil-fed male rats had lower levels of Mtor, Mkk4 (*p* ≤ 0.06), and p-Smad3/Smad3 (*p* ≤ 0.05) than female offspring of lard-fed, but not control diet-fed, male rats (Fig. 6). There was no difference (*p* > 0.05) between female offspring of control diet- and lard-fed male rats regarding these proteins (Fig. 6). In addition to promoting EMT, all these altered proteins are involved in increasing cell survival, growth, migration, and invasion.

## Discussion

Breast cancer is a complex disease with a multifactorial etiology [38]. It is increasingly evident that in utero environment can program later susceptibility to breast cancer [39]. The findings of our present study suggest that breast cancer risk can be determined even earlier through diet-induced changes in paternal germ cells before conception. Our study shows that, compared with female offspring of control diet-fed fathers, offspring of lard-fed fathers did not differ in tumor latency, growth, or multiplicity. However, female offspring of lard-fed fathers had increased elongation of the mammary epithelial tree, number of TEBs, and tumor incidence compared with both control diet- and corn oil-fed fathers, showing that paternal exposure to a lard-based high-fat diet containing SFA increased their daughters’ mammary cancer risk. TEBs are considered sites of tumor initiation [40], and increased epithelial elongation reflects rapid epithelial growth [41]. Additionally, female offspring of lard-fed fathers showed increased cell proliferation and decreased apoptosis in the mammary gland lobules compared with female offspring of both control diet- and corn oil-fed fathers. Altogether, these findings support the view that altered mammary gland development represents a potential underlying mechanism of increased breast cancer risk [42].

Compared with female offspring of control diet-fed fathers, female offspring of corn oil-fed fathers had decreased tumor growth. There was no difference in tumor incidence, latency, or multiplicity between female offspring of control diet- and corn oil-fed fathers. In addition, female offspring of corn oil-fed fathers had longer tumor latency, decreased tumor growth, and decreased multiplicity compared with female offspring of lard-fed fathers. These data show that paternal exposure to a corn oil-based high-fat diet containing n-6 PUFA had an effect opposite that of a lard-based high-fat diet and reduced their daughters’ mammary cancer risk.

Although male rats that were fed the lard-based and corn oil-based high-fat diets consumed the same amount of calories, lard increased the body weight and size of epididymal fat pads more than corn oil did. Thus, different fatty acids can have distinct effects on adipose accumulation, as already shown by others [43]. Our results further show that lard, but not corn oil, elicited detrimental effects on male reproductive parameters (fewer normal sperm cells and lower daily sperm production). This is in line with earlier human and animal data showing that SFA disrupt testicular metabolism and sperm quality, while PUFA are essential for sperm cell membrane fluidity and flexibility as well as fertilization [44]. Excessive epididymal fat in lard-fed males may have been detrimental to spermatogenesis, as epididymal tissue is an essential depot for spermatogenesis [45]. The adverse effects of lard may not be mediated through increased insulin resistance. Although a correlation between insulin resistance and impaired sperm production has been reported in rats fed a diet high in SFA [46], as also found in the present study, a corn oil-based high-fat diet also impaired insulin tolerance but did not affect male reproductive parameters. We propose that impaired sperm quality and function in lard-fed fathers could be associated with disruption in metabolic programming and increased breast cancer risk among their daughters.

The impact of obesity in fathers leading to metabolic dysfunction in their female offspring was previously observed in rodent studies [10], and it was also seen in our present study. Female offspring of both lard- and corn oil-fed fathers exhibited increased body weight and adiposity. However, only female offspring of lard-fed fathers displayed an impaired insulin response, indicating that the type of dietary fatty acids consumed represents a key factor in metabolic programming through the male germline.

Epigenetic modifications that are necessary for achieving reproductive capacity of male gametes include DNA methylation, histone retention, and expression of noncoding RNAs such as miRNAs [47]. Because miRNAs can modulate the expression of hundreds of mRNAs that affect embryonic development as well as the establishment of the offspring’s epigenome [48], they have been proposed to mediate paternal programming effects on the offspring [49]. The epididymis has been implicated as the site of the alterations of miRNA signatures occurring during the maturation of sperm cells, and therefore an increase in epididymal fat pad size could potentially impact inheritance of miRNA signatures and/or the developmental trajectory of the offspring [50]. The impact of high-fat-diet-induced male obesity on the miRNA profile in mature spermatozoa has been examined in rodent studies [51]. In a study by Fullston et al. [52], males fed a high-saturated-fat diet exhibited changes in miRNAs in the testes and mature spermatozoa that target mRNA associated with spermatogenesis, embryonic development, and metabolic diseases in the offspring. We provide further evidence that paternal nutrition can impact the sperm miRNA profile and possibly the subsequent mammary gland miRNA profile, which in turn targets genes implicated in breast cancer and other diseases.

Some of the miRNAs that were differentially expressed in the lard- and corn oil-fed fathers’ germ cells also were differentially expressed in their daughters’ mammary glands, although the daughters were never directly exposed to the high-fat diets. When we compared lard-fed fathers and their daughters, we observed three miRNA that were significantly altered in both the sperm and mammary glands of corn oil-fed fathers and their daughters: miR-1897-5p, miR-219-1-3p, and miR-376a#. Since miRNAs can modulate gene expression by inhibiting the translation of mRNA or by directing their degradation [53], we focused on determining if the expression of the top potentially targeted proteins (Additional file 1: Table S1) was altered in the daughters of lard- or corn oil-fed fathers. Among them, we highlight Pkd1 and Tgfβr1. PKD1 is a serine/threonine kinase that is expressed in ductal epithelial cells of the mammary gland, maintains the epithelial phenotype, and prevents EMT [54]. Inhibition of PKD1 can lead to pathological conditions such as cancer [55]. Thus, our finding of increased Pkd1 levels in the mammary glands of corn oil-fed fathers’ offspring, compared with female offspring of lard-fed fathers, is in line with their lowest susceptibility to breast cancer. In addition, compared with female offspring of control diet- and corn oil-fed fathers, another miRNA target, Tgfβr1, was increased in the daughters of lard-fed fathers that displayed the highest susceptibility to mammary cancer. Tgfβr1 expression is related to promotion of breast carcinogenesis through multiple mechanisms, including enhancing EMT [56]. We assessed the protein levels of up- and downstream signaling partners of Tgfβr1. Female offspring of lard-fed fathers showed higher protein levels of Tgfβ than female offspring of both control diet- and corn oil-fed fathers, as well as higher protein levels of Akt and p-Jnk than control diet-fed fathers. In addition, female offspring of corn oil-fed rats had lower levels of Mtor, Mkk4, and p-Smad3/Smad3 than female offspring of lard-fed fathers. These proteins collectively play important roles in cell survival, growth, migration, and invasion [57, 58]. These findings indicate that mechanisms other than miRNAs contribute to changes in gene expression in the daughters’ mammary tissue following paternal exposure to a lard-based high-fat diet.

Because fathers, mothers, and their daughters tend to share the same nutritional habits [59], it is important to further investigate if paternally programmed breast cancer risk is affected by maternal and female offspring’s fat intake. Maternal intake of a high corn oil diet during pregnancy increases female offspring’s mammary cancer risk [8], while intake of lard has opposite effects [9]. In addition, because obesity-induced altered sperm miRNA expression in the fathers can be normalized through exercise or dietary intervention (consumption of balanced diet), which then improves the metabolic health of female offspring [60], the efficacy of a similar intervention in reducing daughters’ breast cancer risk should be investigated.

## Conclusions

In the present study, we show that paternal intake of a lard-based high-fat diet rich in SFA increases female offspring’s mammary cancer risk, as indicated by the increased elongation of the mammary epithelial tree, number of TEBs, and tumor incidence in female offspring of lard-fed fathers compared with female offspring of both control diet- and corn oil-fed rats. However, if the paternal fat source is corn oil that is high in n-6 PUFA, these male rats’ offspring’s mammary cancer risk is reduced, as indicated by decreased tumor growth in female offspring of corn oil-fed fathers compared with female offspring of both control diet- and lard-fed fathers, as well as by longer tumor latency and decreased tumor multiplicity compared with female offspring of lard-fed fathers. Altered miRNA expression in fathers’ sperm and daughters’ mammary glands may at least underlie these effects, but other epigenetic changes are likely to be involved. Our findings highlight the importance of paternal nutrition in affecting future generations’ risk of developing breast cancer.

### Abbreviations

Akt, v-akt murine thymoma viral oncogene; ANOVA, analysis of variance; Casp3, caspase 3; CB, rats that were fed a corn oil-based high-fat diet and their offspring; Cebpβ, CCAAT/enhancer-binding protein beta; Cfl, cofilin; CO, rats that were fed a control diet and their offspring; c-Raf, v-raf leukemia viral oncogene; DMBA, 7,12-dimethylbenz[a]anthracene; EMT, epithelial-to-mesenchymal transition; Erk1/2, extracellular signal-regulated kinase 1/2; Igf1r, insulin-like growth factor 1 receptor; ITT, insulin tolerance test; LB, rats that were fed a lard-based high-fat diet and their offspring; LSD, least significant difference; miRNA or miR, microRNA; Mkk4, mitogen-activated protein kinase kinase 4; mRNA, messenger RNA; Mtor, mechanistic target of rapamycin; MUFA, monounsaturated fatty acid; p38, mitogen-activated protein kinase 14; p-Jnk, phosphorylated mitogen-activated protein kinase 8; Pkd1, protein kinase D1; p-Smad3/Smad3, phosphorylated Smad family member 3/Smad family member 3 ratio; PTEN, phosphatase and tensin homolog; PUFA, polyunsaturated fatty acid; Ras, Harvey rat sarcoma virus oncogene; SCLB, somatic cell lysis buffer; SFA, saturated fatty acid; TEB, terminal end bud; Tgfβr1, transforming growth factor, beta receptor I

## Additional file

Additional file 1: Table S1. Canonical IPA analyses of the target pathways and molecules modulated by altered miRNA from father’s sperm and 50-day-old female offspring mammary glands from lard-fed (LB) and corn oil-fed (CB) males. (DOC 31 kb)
