# Supplementary material for: Paternal programming of breast cancer risk in daughters in a rat model: opposing effects of animal- and plant-based high-fat diets
Source: Breast Cancer Res. 2016 Jul 26;18:71. doi: 10.1186/s13058-016-0729-x (PMC4960664; doi:10.1186/s13058-016-0729-x)
Supplement: Additional file 1: Table S1. — Canonical IPA analyses of the target pathways and molecules modulated by altered miRNA from father’s sperm and 50-day-old female offspring mammary glands from lard-fed (LB) and corn oil-fed (CB) males. (DOC 31 kb) [file 13058_2016_729_MOESM1_ESM.doc]

**Additional file 1: Table S1**: Ingenuity Canonical Pathway analyses of the target pathways and molecules modulated by altered miRNA from father’s sperm and 50 days old female offspring mammary gland from lard (LB) and corn oil (CB) fed males.

| **Ingenuity Canonical Pathways** | **-log (p-value)** | **Ratio** | **Molecules** |
| --- | --- | --- | --- |
| Prolactin Signaling | 4.62E00 | 9.59E-02 | PRKCI,SP1,PIK3R1,PRKCE,SOCS4,**CEBPB**,**PRKD1** |
| PTEN Signaling | 4.11E00 | 6.78E-02 | **TGFBR1**,**CASP3**,PIK3R1,FGFR1,**IGF1R**,BMPR2,  FOXG1,BCL2L11 |
| Huntington's Disease Signaling | 4.03E00 | 4.78E-02 | GRM5,HDAC9,PRKCI,**CASP3**,SP1,PIK3R1,**IGF1R**,  PRKCE,CDK5R1,SIN3A,**PRKD1** |
| Growth Hormone Signaling | 3.79E00 | 8.7E-02 | PRKCI,PIK3R1,**IGF1R**,PRKCE,SOCS4,**PRKD1** |
| HER-2 Signaling in Breast Cancer | 3.56E00 | 7.89E-02 | PRKCI,PIK3R1,PRKCE,PARD6B,ITGB8,**PRKD1** |
| Role of NFAT in Cardiac Hypertrophy | 3.56E00 | 5.03E-02 | HDAC9,PRKCI,**TGFBR1**,PIK3R1,IGF1R,MEF2A,  PRKCE,**PRKD1**,CABIN1 |
| Type II Diabetes Mellitus Signaling | 3.33E00 | 5.98E-02 | PRKCI,PIK3R1,PRKCE,SOCS4,**CEBPB**,ACSL1,**PRKD1** |
